# Polymorphism at 129 dictates metastable conformations of the human prion protein N-terminal β-sheet†
†Electronic supplementary information (ESI) available. See DOI: 10.1039/c6sc03275c



**DOI:** 10.1039/c6sc03275c

**Published:** 2016-09-30

**Authors:** S. Alexis Paz, Eric Vanden-Eijnden, Cameron F. Abrams

**Affiliations:** a Department of Chemical and Biological Engineering , Drexel University , Philadelphia , PA 19104 , USA . Email: cfa22@drexel.edu; b Courant Institute of Mathematical Sciences , New York University , New York , NY 10012 , USA

## Abstract

We study the thermodynamic stability of the native state of the human prion protein using a new free-energy method, replica-exchange on-the-fly parameterization. This method is designed to overcome hidden-variable sampling limitations to yield nearly error-free free-energy profiles along a conformational coordinate. We confirm that all four (M129V, D178N) polymorphs have a ground-state conformation with three intact β-sheet hydrogen bonds. Additionally, they are observed to have distinct metastabilities determined by the side-chain at position 129. We rationalize these findings with reference to the prion “strain” hypothesis, which links the variety of transmissible spongiform encephalopathy phenotypes to conformationally distinct infectious prion forms and classifies distinct phenotypes of sporadic Creutzfeldt-Jakob disease based solely on the 129 polymorphism. Because such metastable structures are not easily observed in structural experiments, our approach could potentially provide new insights into the conformational origins of prion diseases and other pathologies arising from protein misfolding and aggregation.

## Abstract 2

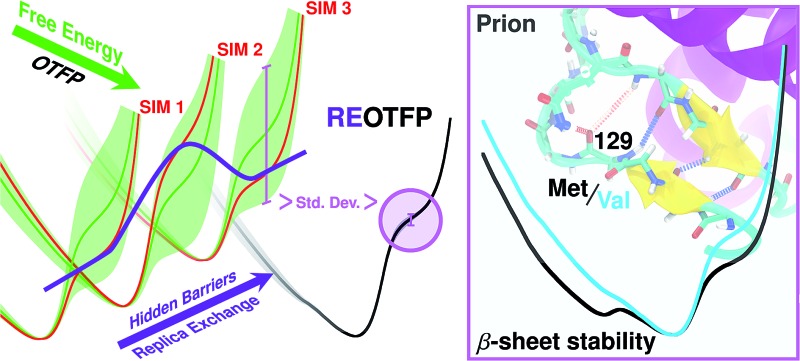
We study the thermodynamic stability of the native state of the human prion protein using a new free-energy method, replica-exchange on-the-fly parameterization.

## 1 Introduction

Transmissible spongiform encephalopathies (TSE's) are a collection of neurodegenerative diseases associated with the misfolding and aggregation of the prion protein (PrP). It is widely accepted that the key phenomenon in pathogenesis is the isomerization of the mostly α-helical native cellular protein (PrP^C^) into a β-sheet-rich misfolded conformation termed “scrapie” (PrP^Sc^).^1^ The “protein-only” hypothesis maintains that this transformation is catalyzed only by PrP^Sc^, which underlies the transmissible nature of prion diseases. However, there is an unusually large spectrum of TSE phenotypes, even within the same animal species, which is difficult to reconcile with the existence of a single PrP^Sc^ state. It has therefore been hypothesized that TSE phenotypic differences arise from different PrP^Sc^ strains which are primarily marked by conformational differences.^2–9^

A methionine–valine polymorphism observed in codon 129 of the PRPN gene has a strong influence on susceptibility to human prion diseases.^10–12^ Clinical aspects and neuropathology of sporadic Creutzfeldt-Jakob disease (sCJD), which represents ∼75% of all prion disease cases,^8,13,14^ significantly vary depending on whether the patient is homozygous for methionine (MM), for valine (VV) or heterozygous (MV). The 129 polymorphism has acquired a central role in attempts to develop early diagnostic^15,16^ and phenotypic classification^6,7,9,17^ of sCJD. This suggests that residue 129 somehow controls which prion strains emerge in sCJD cases.^3^ Moreover, to date all confirmed cases of variant CJD, which, distressingly, is associated with the epidemic of bovine spongiform encephalopathy,^18^ correspond to MM homozygosity at codon 129,^9,19^ and homozygosity also seems to increase susceptibility to iatrogenic CJD.^12,20^ Finally, one of the most striking examples of 129's influence relates to mutation D178N, which causes genetic CJD if residue 129 is valine and Fatal Familial Insomnia (FFI) if residue 129 is methionine.^17,21^

The lack of a structural basis for the apparent correspondence between phenotypic and genotypic variability, especially in relation to residue 129, is a major complication in understanding the prion strain phenomenon. To begin to provide such structural insight, we focus in the contribution of the small antiparallel β strands of PrP^C^. This structure is of particular interest for two reasons. First, the β_1_ strand begins precisely at residue 129. Second, there are at least two conformational changes to PrP^C^ that could conceivably lead to the β-sheet-driven aggregation characteristic of TSE's: (i) elongation of the loop anchored by the β_1_ and β_2_ strands to form a β-hairpin that could attach to similar hairpins in other protomers,^22^ and (ii) breaking of the two β strands to enable either (a) a registry of H-bonds in the β strand distinct from that in native PrP^C^ or (b) interprotomer β-sheet assembly and further aggregation.^23^ In this “PrP^C^-breaking” hypothesis, the small PrP^C^ β-sheet represents an energy barrier for PrP^C^ misfolding and aggregation.

This work is based on the idea that conformational differences among difficult-to-observe metastable intermediates on the PrP^C^ → PrP^Sc^ pathway could underlie 129's multifaceted influence. We explore this idea by direct measurement of the thermodynamics associated with the PrP^C^ β-sheet in all-atom explicit-solvent molecular dynamics (MD) simulations. In particular, we compute the free energy along a collective variable (CV) that measures the degree of “intactness” of the set of four hydrogen bonds that define the PrP^C^ β sheet.^24,25^ Like all CV's, this one is chosen to represent a process that would never spontaneously occur on limited MD timescales at any physically relevant temperature, so a direct CV-biasing method is required. Importantly, however, we show that thermodynamically comparing the mutants associated with the 129/178 polymorphs requires much higher precision (≪1 kcal mol^–1^) than is typically available in existing free-energy methods, due to the common problem of “hidden variables”;^26,27^ *i.e.*, variables not explicitly biased in the calculation are not ergodically sampled and therefore stymie sampling of the CV of interest. To meet this challenge in the study of PrP^C^, we report here the development of a new free-energy method termed replica-exchange on-the-fly free-energy parameterization (RE-OTFP), which seems uniquely effective at overcoming the hidden-variable problem and is able to produce free-energy profiles of high-enough precision to distinguish roles played by point mutations in the stability of the PrP^C^ β-sheet.

This paper is organized as follows. We first present RE-OTFP in context of current methods of CV-biasing in order to explain its advantages. As calibration, we first report its performance on measuring the free energy of the β sheet in the mouse PrP^C^ relative to other methods. We then describe the detailed simulation protocols used to simulate the four 129/178 double-mutants of human PrP^C^ (HuPrP^C^). We then present and discuss our results, with particular emphasis on the role of 129 in the determination of whether a β-sheet breaking or elongation conformational phenotype is preferred.

## 2 Methods

### 2.1 Replica exchange on-the-fly parameterization

CV's are a standard way to simplify the description of complex systems. Free-energy minima in CV space correspond to metastable states, and working in CV space therefore provides a framework for identifying such states for further characterization. Projecting the 3N-dimensional configuration space of the N-atom system ***x*** ∈ ℝ^3N^ onto a chosen lower-dimensional CV space ***z*** ∈ ℝ^M^ *via* mapping functions ***θ***: ***x*** ↦ ℝ^M^ gives rise to a free-energy landscape (FEL) given by1
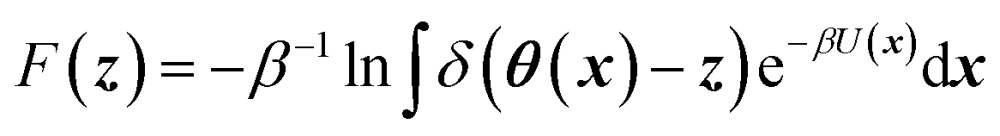
where *U*(***x***) is the potential energy and *β* = (*k*_B_ *T*)^–1^. In general, the computation of the FEL can be extremely expensive and many methods have been devised over the years to implement such a calculation.^28^ CV biasing approaches are drastically limited in accuracy and precision due to the common presence of potential-energy barriers on variables not included in the CV space. A major class of methods meant to overcome this hidden-barriers problem are the so-called “orthogonal-space sampling” approaches.^29–31^ However, these methods can only overcome barriers in hidden variables that are strongly coupled with the dynamic of the CVs, and further assumptions on the system are required.^32^ Another approach has been to harness the non-CV-based enhanced sampling approach of parallel tempering (or, more generally, replica-exchange (RE))^33–35^ in combination with a CV-based method, such as well-tempered metadynamics (WTMD)^24,36^ to generate the composite method “PT-WTMD”, in which the enhanced sampling of variables other than the CV diminishes the likelihood of hidden-variable-based sampling restrictions.^37,38^ However, popular “adaptive” methods like WTMD and the adaptive-biasing force method^39,40^ are particularly sensitive to hidden variables because their adaptivity makes them “remember” sampling limitations these variables cause.^25^ This makes non-adaptive methods, such as on-the-fly free-energy parameterization (OTFP)^25,41^ based on temperature-accelerated MD (TAMD)^42,43^ preferable. Here we make a brief presentation of OTFP/TAMD.

TAMD introduces auxiliary variables ***z*** into an MD simulation, and these are tethered to ***θ***(***x***) *via* harmonic springs, such that 
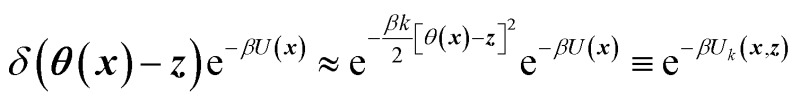
. Replacing the delta-function in eqn (1) with the Gaussian factor defines the mollified estimate of the free energy:2
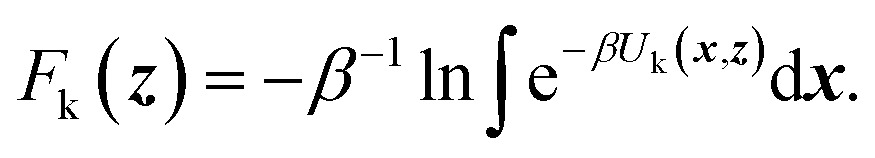



The equations of motion of ***z*** are tuned such they feel negative gradients in *F*_k_(***z***) as if they were conservative forces; providing the ***z*** with a higher “auxiliary” temperature *β̄* ^–1^ > *β* ^–1^ gives them the ability to overcome free-energy barriers and therefore better sample CV space than would a projection of any finite thermal MD simulation. OTFP accumulates the estimators of ∇*F*(***z***) in a TAMD simulation and, positing a basis-function expansion of *F*(***z***), uses them to find optimal coefficients in that expansion, providing an estimate for *F*(***z***). That is, TAMD ensures good sampling of CV space by overcoming free-energy barriers and OTFP actually computes the FEL.^41^ A key feature in the efficiency of OTFP is that estimates of local free-energy gradients converge more quickly than does the uniformity of sampling of CV-space.

Replica-exchange molecular dynamics (REMD) can be implemented on top of TAMD in the following way. Consider an artificial system built up of *R* replicas of a TAMD system, each replica with its own real and auxiliary temperatures. Replicas are labeled following the convention *β*_1_ > *β*_2_ > … > *β*_*R*_ and the auxiliary temperatures can be taken arbitrarily. Let *ρ*(***x***_1_, ***z***_1_, …, ***x***_*R*_, ***z***_*R*_) denote the joint distribution of the *R* replicas. It is given by3
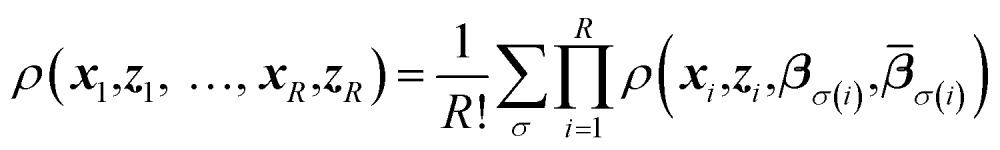
here *σ* denotes permutation over the *R* indices, and we defined

4 *ρ*(***x***,***z***,*β*,*β̄*) = *ρ*(***x***|***z***,*β*)*ρ*(***z***,*β*,*β̄*)

where5
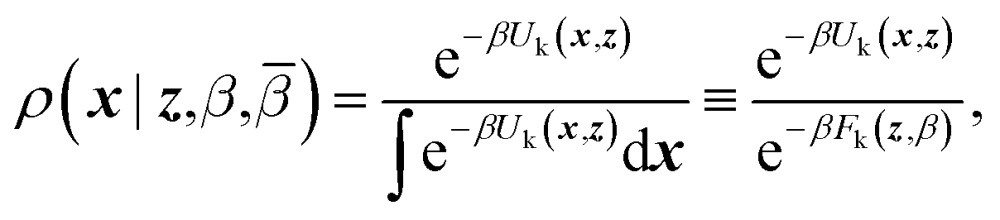
and6
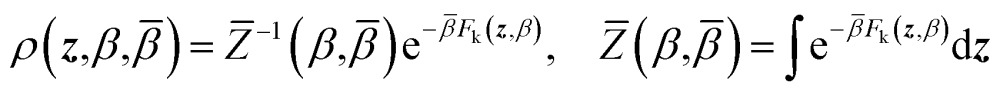



 Eqn (4) says that the ***x*** variables are effectively at equilibrium with (*i.e.*, adiabatically slaved to) the ***z*** variables and feel the temperature *β*, whereas the variables ***z*** navigate on the mollified free energy computed at temperature *β*, *F*_k_(***z***,*β*), but feel the auxiliary temperature *β̄*. Assume now that we attempt to exchange the temperatures between replica *i* having positions (***x***_*i*_,***z***_*i*_) and temperatures (*β*_*i*_,*β̄*_*i*_), and replica *j* having positions (***x***_*j*_, ***z***_*j*_) and temperatures (*β*_*j*_,*β̄*_*j*_), so that, if the move is accepted, immediately after this move replica *i* is now at temperatures (*β*_*j*_,*β̄*_*j*_) and replica *j* at temperatures (*β*_*i*_,*β̄*_*i*_), with their respective positions unchanged. The metropolis acceptance probability of this move is7
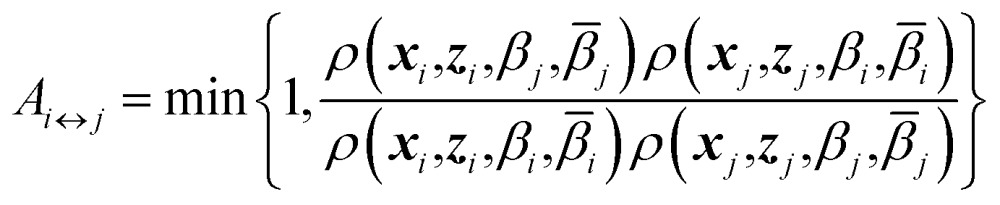



Using (4), the factor involving ratios of *ρ*'s can be written explicitly

8 *A*_*i*↔*j*_ = min{1,exp(*Δ*_*i*,*j*_)}

where

9 *Δ*_*i*,*j*_ = (*β*_*j*_ – *β*_*i*_)(*U*_k_(***x***_*j*_,***z***_*j*_) – *U*_k_(***x***_*i*_,***z***_*i*_)) + (*β̄*_*j*_ – *β*_*j*_)(*F*_k_(***z***_*j*_,*β*_*j*_) – *F*_k_(***z***_*i*_,*β*_*j*_)) + (*β̄*_*i*_ – *β*_*i*_)(*F*_k_(***z***_*i*_,*β*_*i*_) – *F*_k_(***z***_*j*_,*β*_*i*_))

As can be appreciated from eqn (8), the acceptance criterion requires as input the free energies at the two replica temperatures, *F*(***z***,*β*_*i*_) and *F*(***z***,*β*_*j*_), each evaluated at both points in ***z***-space, ***z***_*j*_ and ***z***_*i*_. We propose here to use OTFP to estimate the free energy differences needed in the acceptance criterion. As the simulation progresses, the computed FEL of each replica will be continuously refined *via* OTFP.

Exchange moves between adjacent replicas are attempted at regular intervals. Furthermore, many trials can be performed simultaneously: it is a common practice to consider only neighboring replicas for exchange which allows ∼*R*/2 independent simultaneous attempts. In each attempt, the (groups of) processors running the *i*th and *j*th replica communicate to each other their instantaneous values of ***z***_*i*_ and ***z***_*j*_ respectively. Then, each processor can compute the values of *F*(***z***_*j*_,*β*_*i*_) – *F*(***z***_*i*_,*β*_*i*_) and *F*(***z***_*j*_,*β*_*j*_) – *F*(***z***_*i*_,*β*_*j*_) as needed. One of the processors communicates back the value obtained and its instantaneous value of *U*_k_(***x***,***z***). The other processor will use this information to compute if the exchange is accepted using eqn (8). If is accepted, only the real and auxiliary temperatures are swapped. Finally, the momenta associated with ***x***_*i*_ and ***x***_*j*_ are rescaled using the factors 
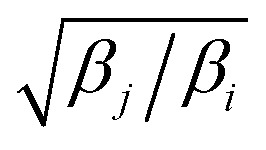
 and 
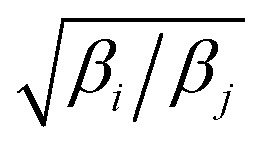
 respectively.^44^ Since we evolve the auxiliary variables using Brownian dynamics, there is no momentum associated with them. However, if different stochastic dynamics are used, equivalent scaling factors using the auxiliary temperatures should be applied.

We have given the name “replica-exchange on-the-fly free-energy parameterization” (RE-OTFP) to the present combination of REMD and TAMD with OTFP. Note that the RE-OTFP has similarities with PT-WTMD.^37^ This becomes evident in the comparison of the acceptance rules for both methods: it is possible to shown that the two terms with the free-energy differences in eqn (9) are equivalent to the two bias terms in the acceptance rule of PT-WTMD. The analogy between RE-OTFP and PT-WTMD is not surprising, considering the similarities between OTFP and WTMD pointed out in our previous work.^25^ Note, however, that there is no history-dependent bias in RE-OTFP.

### 2.2 Simulation set-up and protocols

The number of replicas and their corresponding temperatures are important choices that can drastically affect sampling in a replica exchange simulation. Following Kone and Kofke,^45^ the average acceptance probability can be approximated as10
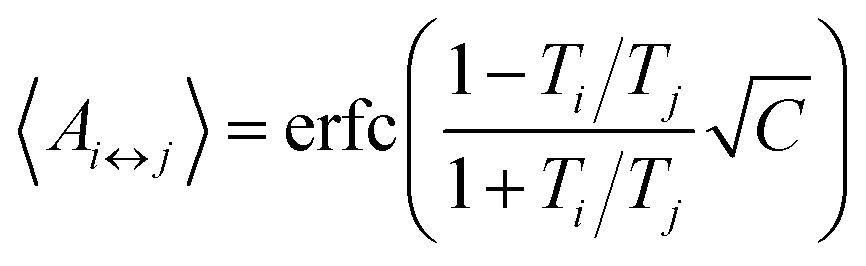
where *C* is the (extensive) heat capacity (in units of the Boltzmann constant), assumed to be constant in the temperature range between the involved replicas. We have computed *C* of the mouse PrP^C^ at different temperatures using 10 molecular dynamic simulations (see ESI†). The average heat capacity obtained is 61 kcal (mol K)^–1^. For the sake of simplicity we take this value as constant for the temperature range studied. With this assumption, we can use eqn (10) to write11
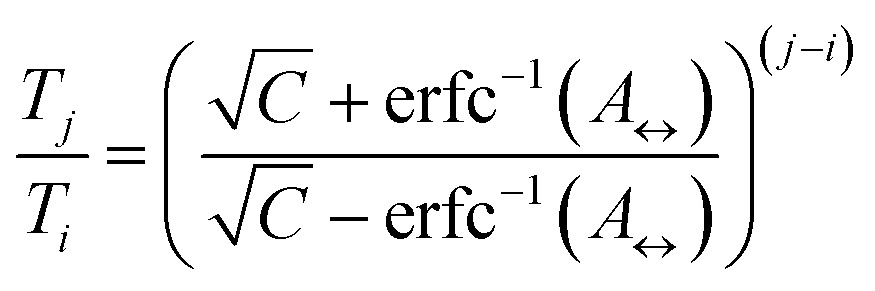
where *A*_↔_ is the average acceptance probability between neighboring replicas. It has been suggested that a value near 0.2 might constitute an optimal set up for this quantity.^45,46^ By fixing *T*_2_ = 300 K and *T*_*N*_ = 400 K we use eqn (11) to predict that a number *N* = 30 replicas is necessary to obtain *A*_↔_ = 0.2. Replica temperatures were fixed using the same equation.

Another fundamental factor in the success of an RE-OTFP simulation is the choice of the auxiliary temperatures for each replica. This parameter defines the acceleration strength of each TAMD replica and will also affect the acceptance probability of the exchange between them (eqn (8)). In the present study we choose a constant auxiliary temperature *T*_*i*_ = 3000 K for all replicas. This is the same auxiliary temperature using in our previous work,^25^ which means that the replica here at 300 K will experience the same bias as in that work. However, other choices for *T*_*i*_ are possible and will be carefully studied in future works.

All simulations presented in this work were performed using NAMD 2.9 (ref. 47) and the CHARMM force field^48^ with CMAP corrections.^49^ The system details as well as the equilibration procedure used to obtain the initial configuration of the mouse prion protein (MoPrP) are described elsewhere.^25^ An analogous protocol was used for the human prion protein HuPrP-; 129M: after solvating the NMR structure^50^ (PDB ID: ; 1HJM), 110 ns of equilibration were run while keeping the protein fixed during the first 5 ns. Mutant D178N-; 129M was constructed by substituting residue 178 in this equilibrated system and was subsequently equilibrated for 50 ns. The 129-valine polymorphs were constructed substituting residue 129 in the HuPrP-; 129M and D178N-; 129M equilibrated structures and were subsequently equilibrated another 50 ns. The details of these preliminary simulations and their RMSDs are included in the ESI.†

RE-OTFP replicas were simulated in the NVT ensemble in agreement with eqn (8). The temperature was controlled using a Langevin thermostat with a damping constant of 50 ps^–1^. We devised a standard protocol for RE-OTFP simulations comprising four stages:

#### Initialization

The FEL is considered unknown and therefore no exchanges are allowed. This stage is used to build the first FEL guess using OTFP.

#### Equilibration

This simulation stage is used to allow the different replicas to decorrelate from the initial state. Exchanges are allowed and the FEL is constantly refined.

#### Reset

At the beginning of this stage the statistics accumulated during the previous stages are forgotten. This is intended to prevent any correlation with non-ergodic sampling that may have arisen in earlier stages. The FEL refinement process starts again but in the background while the replica exchanges are performed using the FEL obtained at the end of the equilibration.

#### Production

The FEL obtained in the reset stage is used to perform the replica exchanges and is continuously refined on-the-fly until the end of the simulation.

All RE-OTFP simulations of the prion protein genotypes use 1 ns of initialization, 8 ns of equilibration and 1 ns in the reset stage. The production stage involve 18 ns for MoPrP system and 28 ns for HuPrP and D178N polymorphs.

### 2.3 Validating RE-OTFP with alanine dipeptide

As a further validation of the present RE-OTFP method, we include in Section 3 of the ESI† the 1-D FEL reconstructions for the *ψ* and *φ* angles of alanine dipeptide in water. The simulation parameters for this system where identical to those used for the prion systems. The obtained profiles were compared with those obtained from 74 ns of pure REMD and showed excellent agreement.

## 3 Results

### 3.1 RE-OTFP performance on mouse Prp^C^

**Fig. 1:**
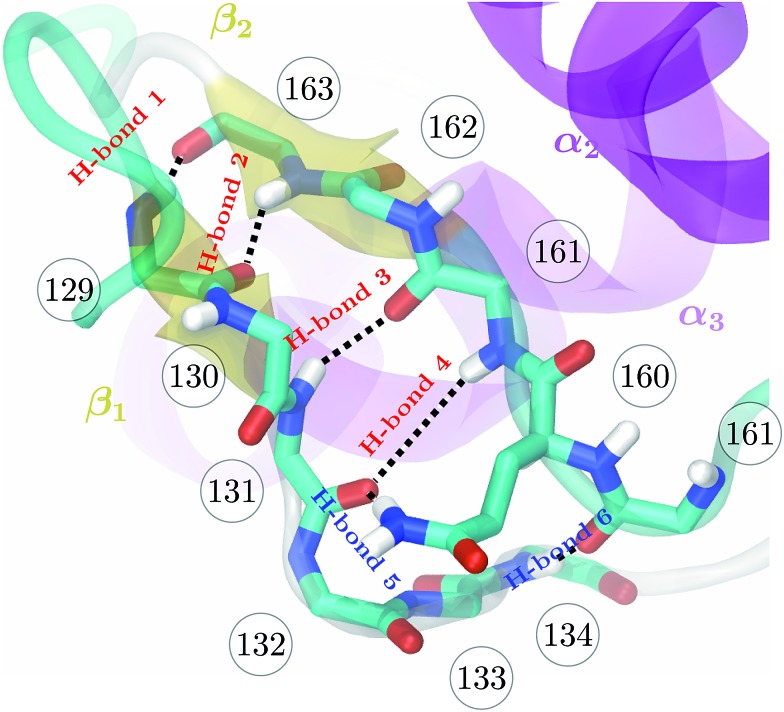
Backbone of the β-sheet structure of the mouse prion protein. Secondary structure, residue numbers and H-bonds labels are included.

We first characterize the performance of RE-OTFP by reconstructing the FEL of the β-sheet structure of the murine prion protein. The CV is12
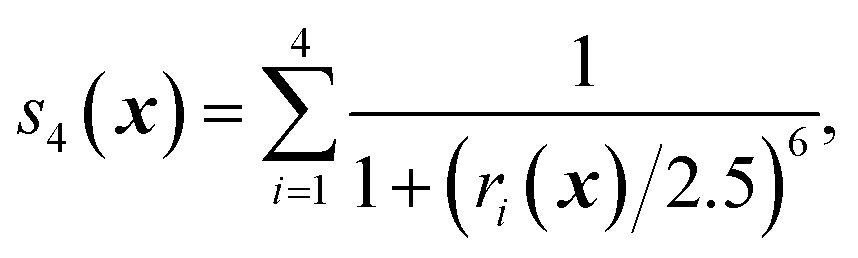
where *r*_*i*_ is the H–N distance of H-bond *i* out of the set of four H-bonds formed between the two short β-strands 129–131 and 161–163. Fig. 1 indicates H-bond 1 to H-bond 4 as well as H-bond 5 and H-bond 6 which are not included in the CV.

**Fig. 2:**
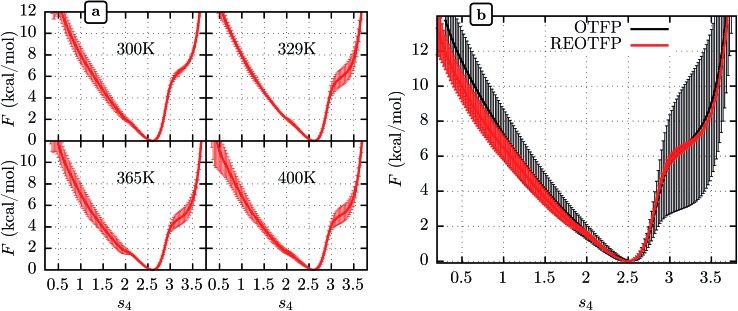
(a) Average free energy landscape (FEL) along *s*_4_ for the MoPrP^C^ at different temperatures obtained *via* RE-OTFP simulations. (b) Comparison between FELs obtained *via* RE-OTFP and pure OTFP.^25^ Error bars correspond to ±one standard deviation from independent simulations (RE-OTFP, *n* = 3; OTFP, *n* = 16).

We performed three independent RE-OTFP simulations, each consisting of 30 replicas spanning from 295 K to 400 K and lasting 28 ns (18 ns of production). The FELs obtained were in excellent agreement among the three independent simulations, as shown in Fig. 2a for four representative temperatures. It is remarkable that this agreement persists even for the free-energy shoulder at *s*_4_ ≈ 3.2, which we showed previously suffers very large errors using WTMD and non-RE OTFP, due to hidden variables.^25^ In Fig. 2b we show a comparison between the FEL obtained at 300 K *via* RE-OTFP and the corresponding profile obtained *via* averaging 16 independent OTFP simulations of 60 ns each from our previous work.^25^ The remarkable precision observed for the RE-OTFP profiles indicates that the method is able to overcome the hidden barriers that underlie error in this region of CV space and thereby achieve high reproducibility. The dispersion of these profiles is small enough to establish that an independent RE-OTFP simulation can adequately represent the average to much less than 1 kcal mol^–1^. Despite the small overhead due to replica exchange, the computational effort for a single RE-OTFP simulation yielding sub-kcal errors is 840 ns (30 × 28), which is somewhat lower than the 960 ns (16 × 60) used to obtain the FEL *via* OTFP with errors of greater than 4 kcal mol^–1^.^25^ We show in the ESI† (Sections 3 and 5) evidence that RE-OTFP outperforms OTFP and REMD separately in both sampling the CV and overcoming hidden-variable sampling issues.

The higher precision obtained *via* RE-OTFP relative to OTFP allows observation of finer details of the FEL's. Fig. 2a shows the averaged FEL for *s*_4_ in the MoPrP^C^ at different temperatures. We observe that the free energy shoulder at *s*_4_ > 3 decreases with increasing temperature. This effect can be explained on the basis of the competition between H-bond 4 and H-bond 5. While the acceptor group for H-bond 4 is in the backbone of residue 131, H-bond 5 involves the side chain of residue 160. When the temperature increases, it is reasonable to expect an increase in the side-chain flexibility and thus H-bond 5 will be weakened, allowing H-bond 4 to form and contribute to *s*_4_. In the opposite direction, Fig. 2 shows the formation of a smaller shoulder near *s*_4_ ≈ 1.8 indicating destabilization of the β-sheet at high temperatures. By inspection of the corresponding configurations, we can relate this shoulder to the full detachment of H-bond 3 leading only H-bond 1 and 2 remaining intact.

### 3.2 Human PrP^C^: effects of 129 and 178 polymorphisms on β-sheet stability

**Fig. 3:**
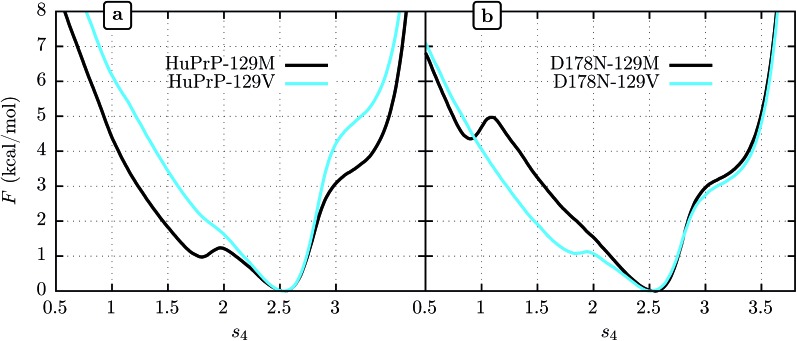
Free energy *vs. s*_4_ for human wild-type PrP^C^ (a) and mutant D178N (b).

We show FEL's at physiological temperature (309 K) for the human PrP^C^ (HuPrP^C^) for both M and V genotypes at position 129 in Fig. 3a. We see that in both cases the thermodynamically most stable state falls around *s*_4_ = 2.5, similar to MoPrP. However, significant differences appear in the free energy of the surrounding subdomains. While the valine genotype displays a FEL similar to that of MoPrP^C^, the methionine genotype shows a striking secondary free-energy minimum at *s*_4_ ≈ 1.8. In Fig. 3b we show the corresponding FEL's for the D178N mutant. Again, the most stable state in all cases is at *s*_4_ = 2.5. With the D178N mutation, however, the 129 polymorphism has the opposite effect on the metastabilities on *s*_4_ relative to WT: the valine genotype shows a secondary minimum at *s*_4_ ≈ 1.8 while the methionine genotype shows no such secondary minimum.

One reasonable interpretation of the secondary minimum at *s*_4_ ≈ 1.8 is as an indicator of a propensity of the β-sheet to unfold.^23^ We see that the valine genotype at 129 therefore stabilizes the β-sheet in the D178 background but destabilizes it in the N178 background. Within this interpretation, a comment must be added in respect to the minimum observed at *s*_4_ ≈ 0.8 in D178N-; 129M (Fig. 3b). While this minimum indicates another metastable state present at low *s*_4_, it has a free energy of 4.5 kcal mol^–1^ above the global minimum, which implies a marginal population of near the 0.3% of the equilibrium configuration at *s*_4_ ≈ 2.5. In contrast, the secondary minima at *s*_4_ ≈ 1.8 present in D178N-129V and HuPrP-; 129M is expected to be more significant in the β-sheet stability because it represent nearly the 20% of the equilibrium configuration.

**Fig. 4:**
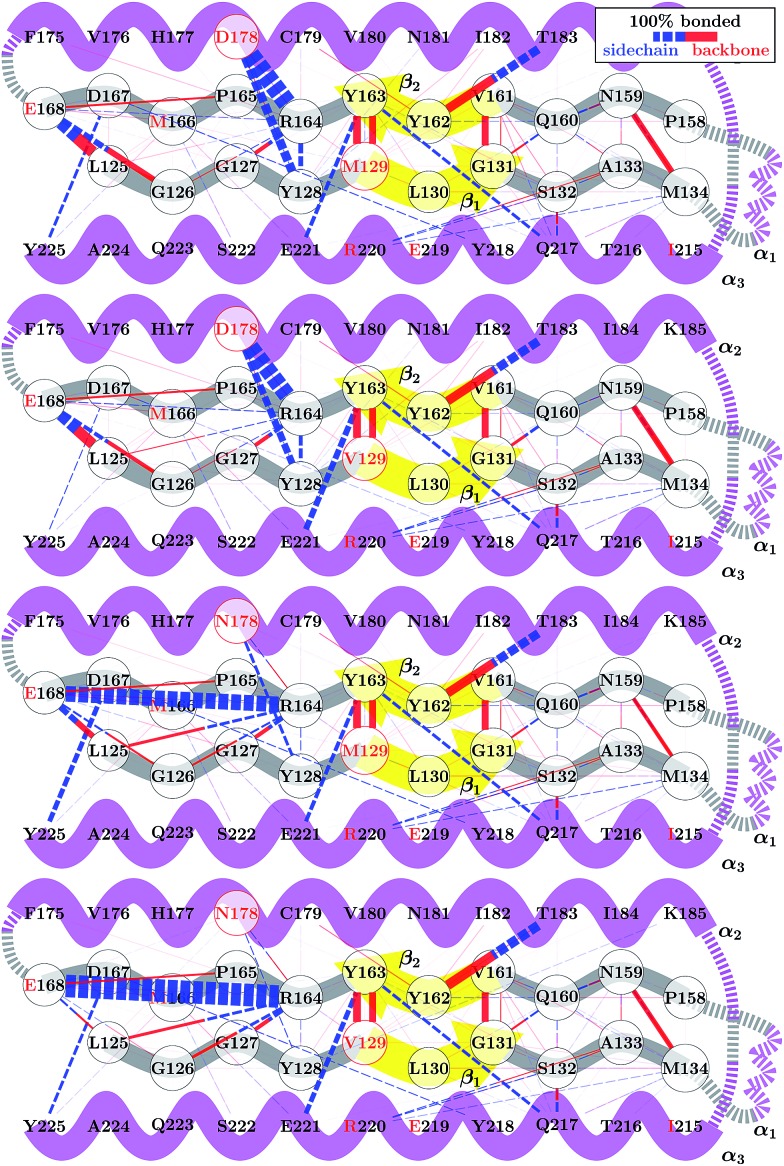
Schematic of the H-bond network near the β-sheet structure of WT HuPrP^C^ and the D178N mutant. Bond thicknesses indicate relative frequencies with which H–O bond lengths below 2.5 Å are observed at 309 K.

Although the 178 polymorphism has an effect on the H-bond network in the ground-state PrP^C^ conformations, an effect of the 129 polymorphism on these states is not apparent. Fig. 4 shows the H-bond network near residue 178 for the equilibrium configurations (*i.e.* 2.4 < *s*_4_ < 2.6) at 309 K from the REOFTP simulations. The different line widths in the schematic represents the frequency at which we observe each H-bond at a bond distance below 2.5 Å. In the wild-type, the side chain of D178 acts as an H-bond donor and bind the side chain of R164 and Y128. After the mutation D178N is introduced, R164 forms a strong H-bond with E168 and no longer interacts with 178. However, no significant differences in the H-bond network were observed between the different 129 genotypes in the D178N mutant or in the wild-type. The same observations remain true also in the H-bond networks for *s*_4_ ≈ 1.8 (see Fig. S11 in the ESI†).

The FEL for the wild-type and mutant polymorphs predicts an equilibrium configuration of *s*_4_ ≈ 2.5 in all cases. This is in agreement with the crystal structures for D178N-M129, D178N-V129 and the three structures for HuPrP-129V reported by Lee *et al.* ^51^ After adding the corresponding hydrogens to these structures we measure values of *s*_4_ = 2.57, 2.52, 2.65, 2.61 and 2.51 respectively. Nonetheless, a detailed comparison between the H-bond network from our simulations, presented in Fig. 4, and networks observed in crystal structures is not straightforward. Lee *et al.* observed several different configurations for the H-bond network surrounding residues 129 and 178, indicating high variability in the side-chain packing for this area.^51^ The present results also resemble those of Hosszu *et al.*,^52^ where the NMR and crystal structure for the two wild type polymorphs are shown to closely match.^52^ Furthermore, Hosszu *et al.* find no significant differences in local dynamics and structural stability in measurements of amide protection factors and chemical shifts, which is consistent with our present picture of the H-bond network.

## 4 Discussion

It has been proposed that conformational changes in the native β-sheet structure may have an important role in the PrP^C^ → PrP^Sc^ conversion. The main reason to focus on this small structure is that this conversion should be accompanied by a large increase in the β-sheet content of PrP^C^. However, two seemingly contrary hypotheses arise from considering the role played by this structure: the β-sheet serves as a seed that guides the elongation as an early stage of the PrP^C^ transformation^22^ or the native β-sheet must break to allow the transformation to occur.^23,53^ The results obtained in the present work motivate us to contribute a different hypothesis to this discussion: crossing an energetic barrier associated first with rupture of the native β-sheet or first with elongation of the native β-sheet each commit PrP^C^ to distinct infectious PrP^Sc^ forms, and the polymorphism at 129 can favor one or the other. The minima observed at *s*_4_ ≈ 1.8 indicate that the methionine genotype seems to be more susceptible to breaking of the native β-sheet structure (Fig. 3). The FEL for the valine genotype indicates an improved native β-sheet stability at physiological temperatures, which is consistent with the idea of an elongation of the native β-sheet structure as a mechanism of conversion.

Modern classification schemes for sporadic CJD phenotypes originated with Parchi *et al.* ^6–8^ In this scheme, two different PrP^Sc^ types are defined, and according to patient allele at codon 129, six sporadic CJD sub-types are identified. These sub-types conform very well to the clinical and histological observations. The evidence suggests that each PrP^Sc^ type arises from different PrP^Sc^ conformations. At the same time, a strong correlation exists between the PrP^Sc^ type and the codon 129 allele. PrP^Sc^ type 1 presents in 95% of the sporadic CJD patients who are MM homozygous, whereas PrP^Sc^ type 2 was present in 86% of the patients who are either VV homozygous or MV heterozygous^5,54^ The idea that each polymorphism can facilitate two different ways of PrP^C^ → PrP^Sc^ interconversion is consistent with these observations. If our hypothesis is correct, we predict that PrP^Sc^ type 1, which correlates with the M genotype, arises from a PrP^C^ → PrP^Sc^ that proceeds by a mechanism initiated by the breaking of the PrP^C^ native β-sheet structure, while PrP^Sc^ type 2 correlates with the V genotype and follows a PrP^C^ → PrP^Sc^ reaction through a native β-sheet elongation mechanism.

In the most simplistic projection of this model, the misfolding of a PrP^C^ through β-sheet breaking should lead to a PrP^Sc^ type 1 that catalyzes further β-sheet breaking. In this approach, is reasonable to consider that the recruiting of PrP^Sc^ type 1 will be significantly affected if many of the PrP^C^ genotypes that reacts are resistant to the β-sheet breaking. The same reasoning applies to the elongation mechanism and PrP^Sc^ type 2. Here we are considering that the 129 allele is only tilting the thermodynamic balance to one or other reaction, thus an unfavorable crossing catalysis might still be possible. The observation that homozygosity is not a fully restrictive condition to prion disease might arise from this consideration.

Our hypothesis postulates the existence of a “fork” in the PrP^C^ → PrP^Sc^ reaction mechanism that can be affected by the polymorphism 129 *via* its influence on the β-sheet structure stability. This idea of course does not clarify the full mechanism of PrP^C^ → PrP^Sc^ reaction and does not gives any direct insight in the PrP^Sc^ aggregation process. Furthermore, besides the classification scheme of Parchi *et al.*, other classification systems exist and more PrP^Sc^ types have been observed,^9,55^ and the existence of several PrP^Sc^ types was considered a potential cause.^3^ Therefore, it is reasonable to consider the possibility of other forks in the PrP^C^ → PrP^Sc^ reaction mechanism,^56^ other thermodynamic/kinetic determinants in the aggregation process^4^ or even the action of other biomolecules in this process. Nevertheless, the results presented here might give a thermodynamic/kinetic explanation to the important influence of residue 129 in the different disease phenotypes and susceptibilities.

## 5 Conclusion

We have reported results of all-atom free-energy calculations assessing the stability of the native N-terminal β-sheet of native human PrP^C^ using the new method of replica-exchange on-the-fly-parameterization (RE-OTFP). We showed unprecedentedly high precision of the free-energy landscapes (≪1 kcal mol^–1^) computed using RE-OTFP and illustrated that RE-OTFP is vastly cheaper on a per-unit-error basis than any current collective-variable biasing free-energy method. We compared free-energy landscapes for the four genotypes arising from the M129V/D178N polymorphs of the human PrP^C^, which were selected due to the singular importance of these two residues in determining variability of TSE phenotypes. Although all landscapes had the same ground state structure, which agrees with experiment for all polymorphs, we showed clear differences among these free-energy landscapes in the form of new metastabilities. This led us to hypothesize that TSE strains might originate from differences in the energetic accessibility of metastable states that select for either elongation or breaking of the native β-sheet on pathways to distinct infectious PrP^Sc^ conformations. The marginal effect of polymorphism 129 in the equilibrium configuration together with the FELs presented indicate that the study of excited PrP^C^ configurations might lead to further insights in the prion phenotype phenomena. Finally, the precision of the FELs generating using RE-OTFP indicate that it might be the method of choice for characterizing thermodynamic stability differences of protein conformations arising from seemingly conservative mutations that do not introduce or destroy any specific interactions but nonetheless lead to distinct functional phenotypes.
