# Supplementary material for: Polymorphism at 129 dictates metastable conformations of the human prion protein N-terminal β-sheet
Source: Chem Sci. 2016 Sep 30;8(2):1225–32. doi: 10.1039/c6sc03275c (PMC5369536; doi:10.1039/c6sc03275c)
Supplement: Supplementary file 1 [file SC-008-C6SC03275C-s001.pdf]

Supporting information for: Polymorphism at 129 dictates metastable conformations of the human prion protein N-terminal β-sheet

S. Alexis Paz,[†] Eric Vanden-Eijnden,[‡] and Cameron F. Abrams[*,†]

†*Department of Chemical and Biological Engineering, Drexel University, Philadelphia, PA 19104, United States.*

‡*Courant Institute of Mathematical Sciences, New York University, New York, NY 10012, United States.*

E-mail: cfa22@drexel.edu

1 Heat-capacity

The heat capacity of MoPrP PrP^C was computed using the variance of the potential energy distribution

$$C_v = \frac{1}{k_B T^2}\left[\langle U^2\rangle - \langle U\rangle^2\right]. \tag{1}$$

The distribution of the potential energy in the simulations together with the computed C_v for MoPrP and alanine dipeptide systems are shown in Figure S1 and S2 respectively.

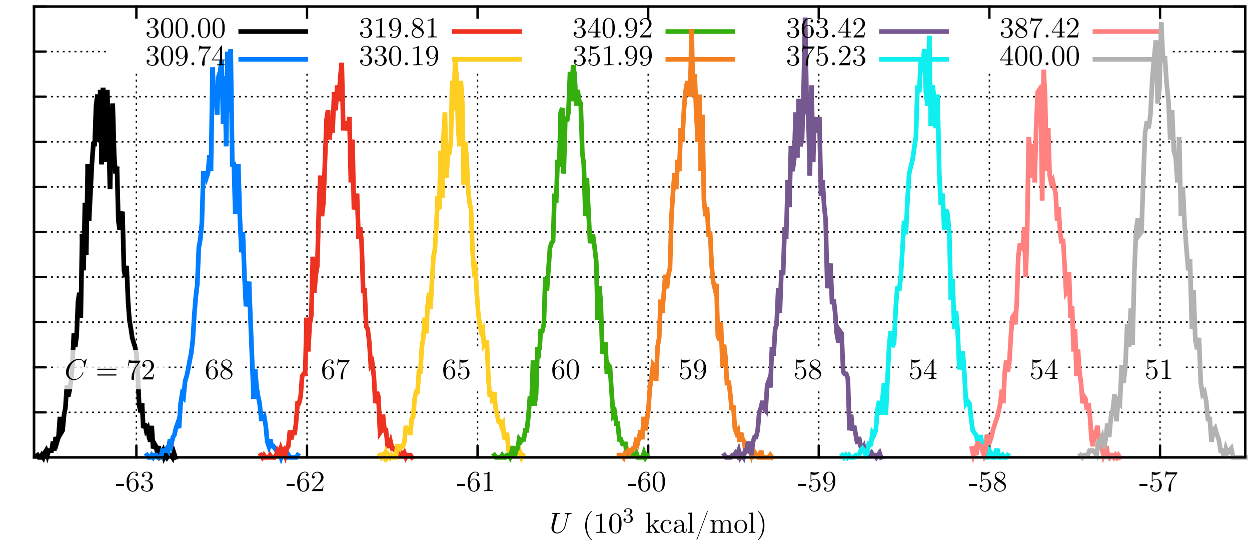

Figure S1: Potential energy distributions and their associated heat capacities for the MoPrP protein. Equation 1 was evaluated using 7 ns MD simulations with constant temperatures from 300 K to 400 K.

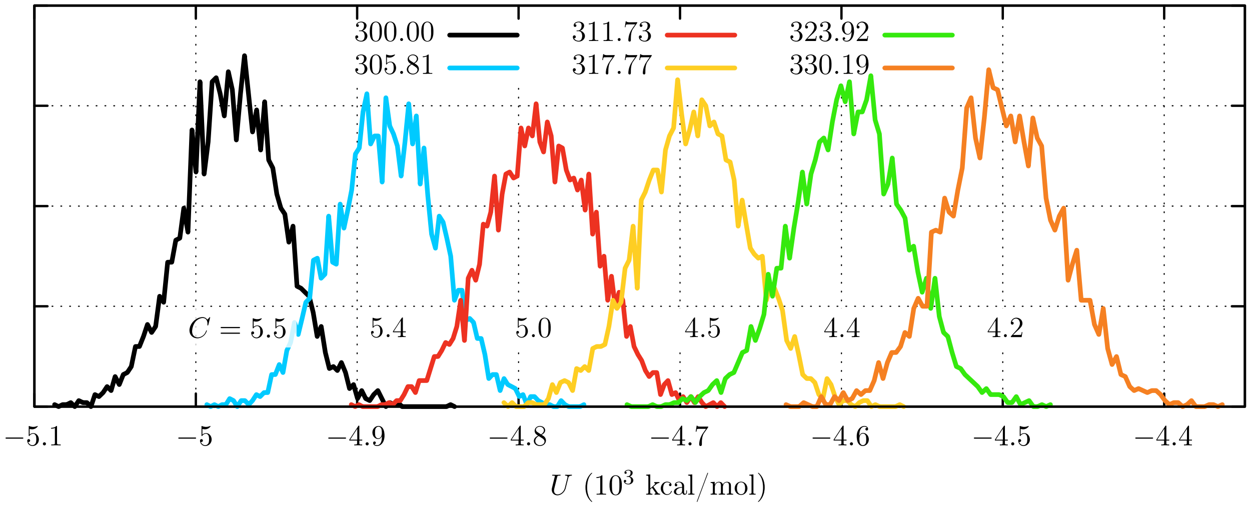

Figure S2: Potential energy distributions and their associated heat capacities for alanine dipeptide. Equation 1 was evaluated using 5 ns MD simulations with constant temperatures spanning 289 K to 400 K

2 Protein equilibration

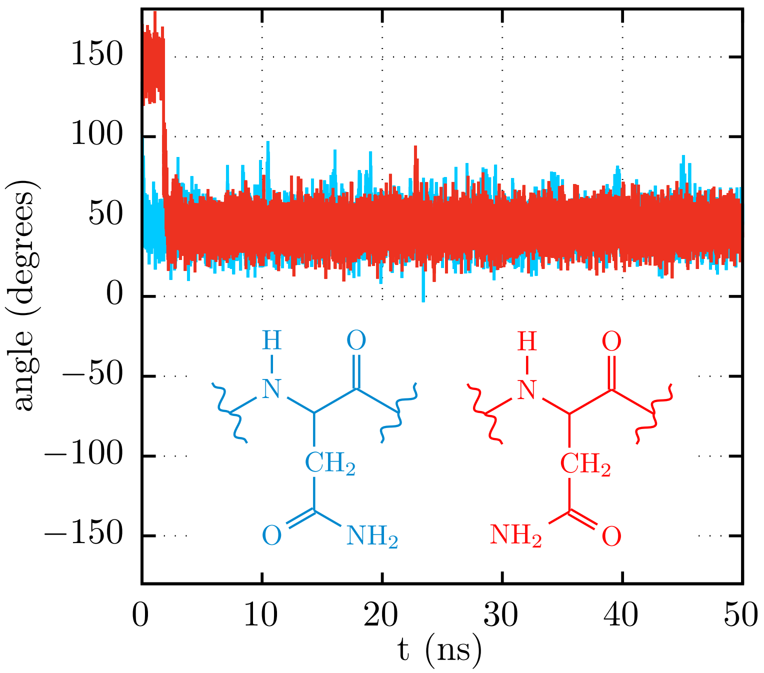

Figure S3: Convergence of the side chain conformations for residue N178 after D178N mutation. The measured angle is the C_β-C_γ-C_δ-O dihedral at the end of the side chain.

For all the protein equilibrations, constant pressure was maintained using a Langevin piston with a constant decay of 50 ps^{-1} and an oscillation time of 100 fs. At the end of the equilibration, the simulation box side length oscillates near 58 Å with standard deviations around 0.06 Å for all the systems. RMSDs for all the equilibration simulations are presented in Figure S4.

The inital configuration of the D178N-129M system was constructed by substituting residue 178 in the equilibrated HuPrP-129M structure. Two possible conformations for the C_β-C_γ-C_δ-O dihedral in the side chain of asparagine are possible for this substitution. Both conformations were separately considered in the equilibration procedure checking that one of them quickly converged to the other. This can be appreciated in Figure S3.

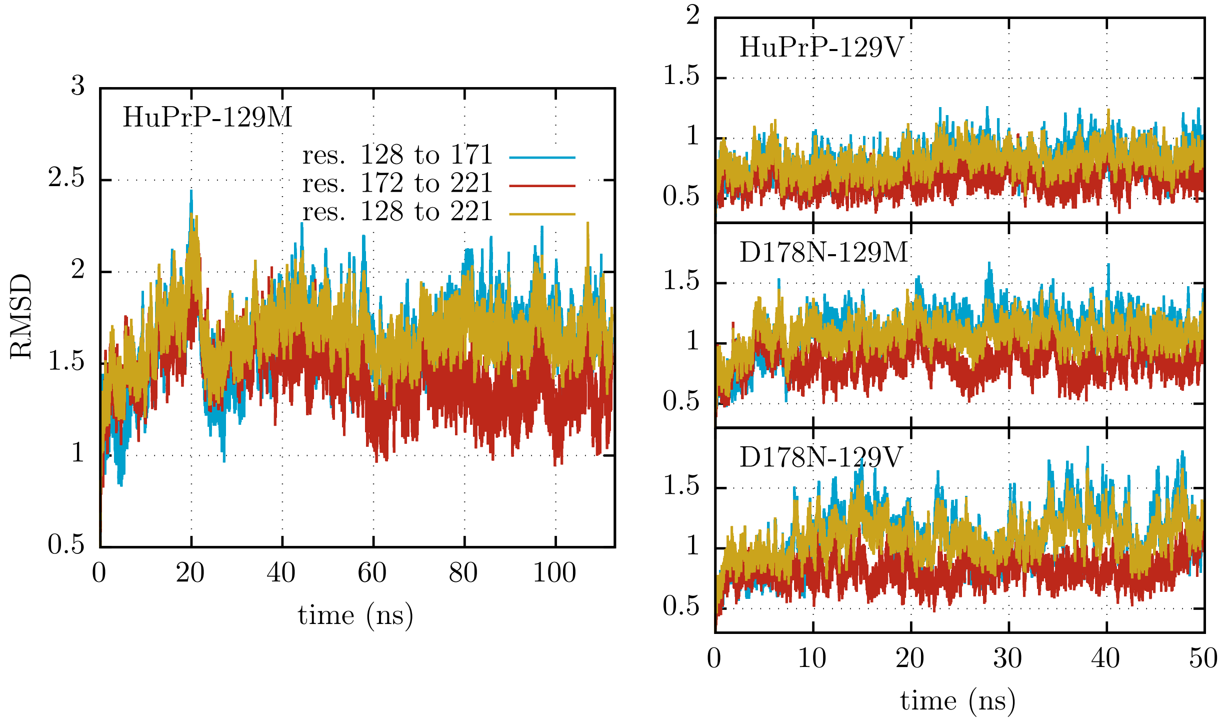

Figure S4: RMSD's for the equilibration of the 4 HuPrP genotypes studied.

3 Validation of RE-OTFP with alanine dipeptide

Alanine dipeptide was solvated with 526 water molecules and the simulation box side length was 24.7 Å after 5 ns of NPT equilibration. The simulation parameters were identical to those used for the prion systems. A 20% acceptance ratio was achieved using 10 replicas spanning 289 K to 400 K. The RE-OTFP simulation time was divided into 500 ps of initialization, 4 ns of equilibration, 500 ps of reset and 25 ns of production. The obtained profiles were compared with those obtained from 74 ns of pure REMD and excellent agreement was found, as can be seen in Figure S5.

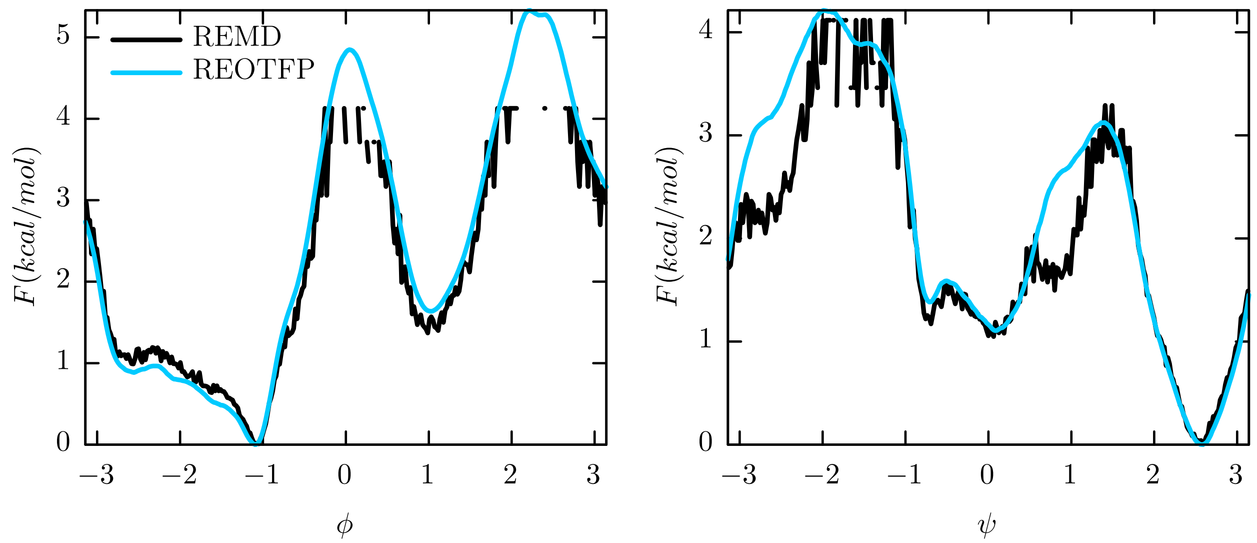

Figure S5: Free-energy profiles for the ϕ and ψ angles of the alanine dipeptide in water computed using RE-OTFP and REMD.

4 Correlation between s_4 and residue 133

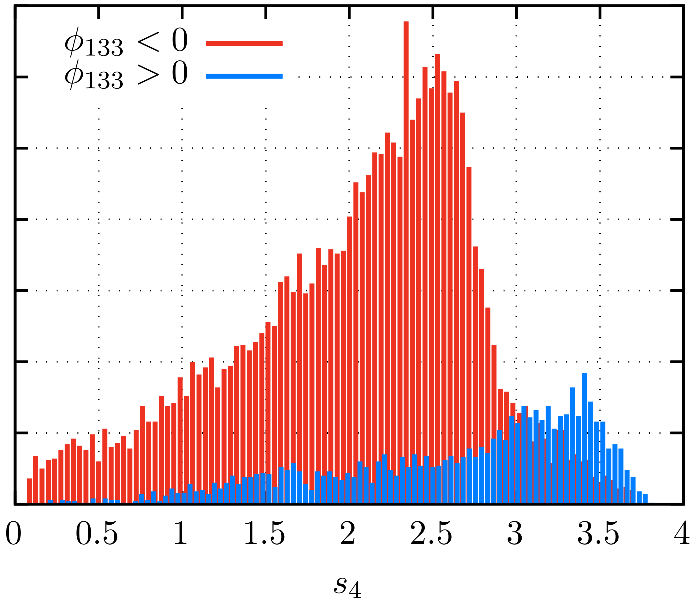

Figure S6: Probability to sample ϕ_{133} near $-\pi/2$ (red) or near $\pi/2$ (blue) for different values of s_4 during a REOTFP simulation (biased).

5 Comparing REOTFP, OTFP and REMD sampling

REOTFP combines a strong acceleration across the chosen CV-space via TAMD (e.g. $\bar{T} =$ 3000K) with a general but less intensive acceleration in the rest of the degrees of freedom via REMD (e.g. $T_N = 400$K). To understand why both kinds of acceleration are needed, we compared the temporal evolution of the CV s_4 (Figure S7a) and the hidden barrier H-bond 5 (Figure S7b) for the different simulation methods. In Figure S7a it is possible to observe that even for the replica with the highest temperature REMD is not able to significantly enhance the exploration of the CV-space. In Figure S7(b) we can see that TAMD is not able to enhance the connection/disconnections events of H-bond 5, because this H-bond is not part of the s_4 variable's definition, while REMD and REOTFP both do.

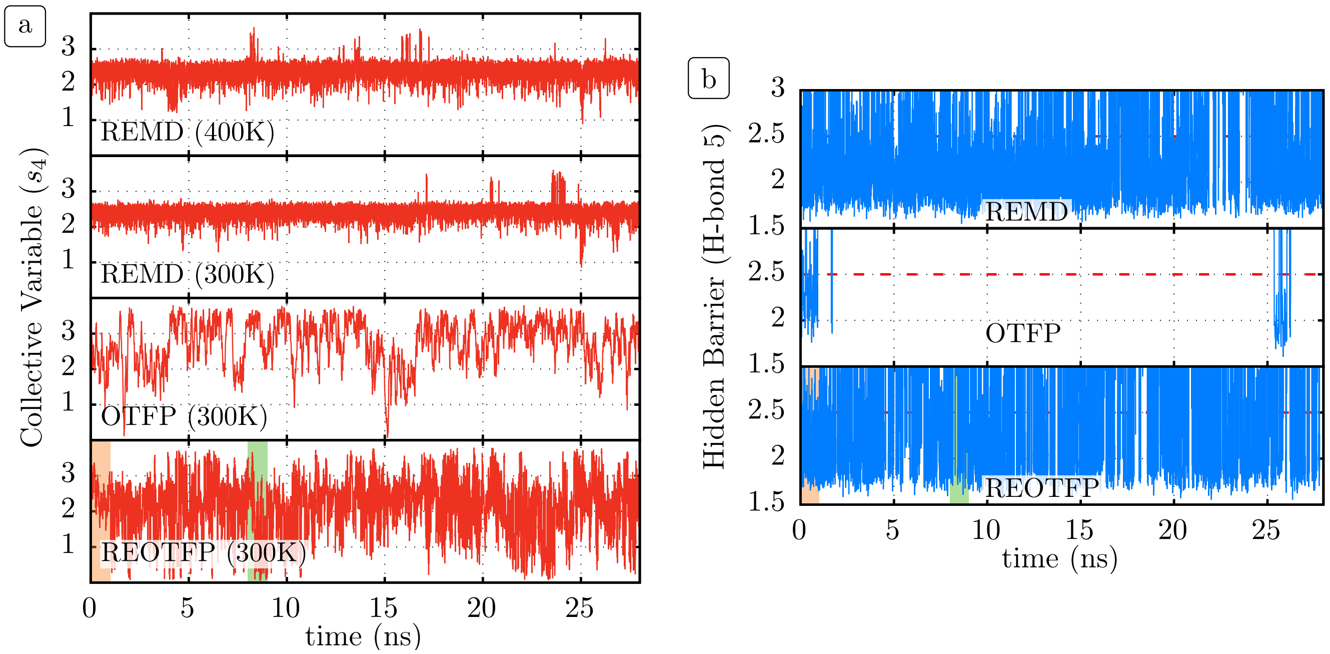

Figure S7: (a) Temporal evolution of the s_4 CV of mPrP^{C} for different simulation methods indicated. Two traces at different temperatures are included for REMD: the highest temperature gives the largest possible exploration for the method. While the same is true for REOTFP, the main contribution of the achieved acceleration is due to the TAMD method. (b) Temporal evolution of H-bond 5 for different simulation methods as indicated. The connection/disconnection of H-bond 5 constitutes a strong hidden barrier for the CV.

Further inspection of Figure S7(a) shows that the sampling obtained via REOTFP for a particular temperature is better than the sampling obtained from a pure TAMD simulation. In REOTFP, the replica TAMD simulations can exchange auxiliary variables resulting in faster diffusion on the CV-space for each temperature.

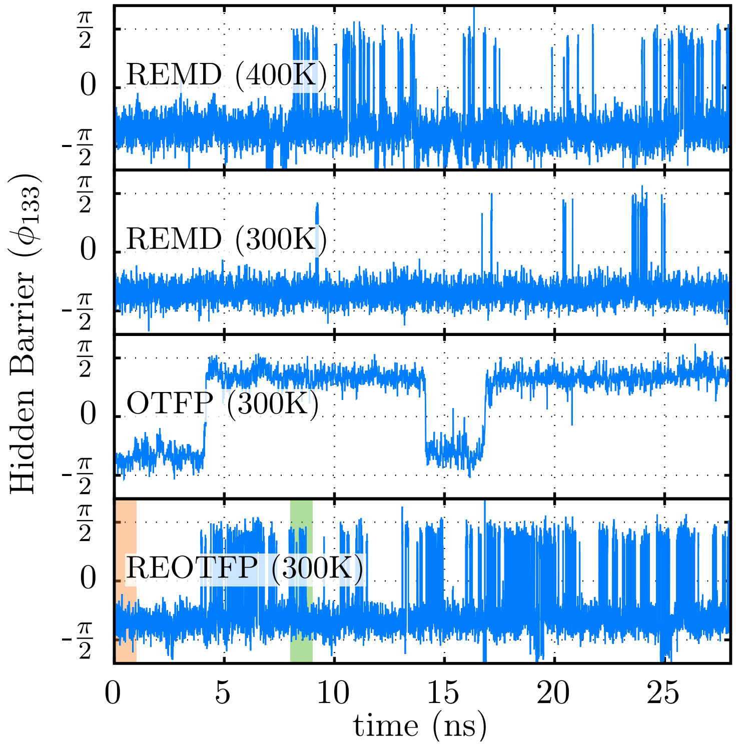

Figure S8: Similar to figure S7a but monitoring the temporal evolution of ϕ_{133}.

Figure S8 shows the temporal evolution of ϕ_{133}, the ϕ backbone dihedral angle of residue 133. We see here that REOTFP can efficiently sample ϕ_{133} at 300K, while the system conformations associated with $\phi_{133} = \pi/2$ are barely sampled by REMD at this temperature. This is because ϕ_{133} and s_4 are strongly correlated; values of ϕ_{133} near $\pi/2$ are more populated when $s_4 > 3$ (see supporting information). REMD alone is not able to efficiently accelerate ϕ_{133} because it cannot access $s_4 > 3$. In this sense, REOTFP can increase the observation frequency of high free-energy configurations and thus facilitate the observation of any others events associated with these configurations, such as ϕ_{133} rotation. In other words, the TAMD component of REOTFP increases the sampling of high-free energy regions of the FEL and

then the REMD component is able to overcome hidden barriers in these regions with needed to specifically address them. This is what enables REOTFP to achieve the reduced error bars observed in the high-free energy regions of the FEL, as was shown in Figure S8.

6 FELs at different temperatures

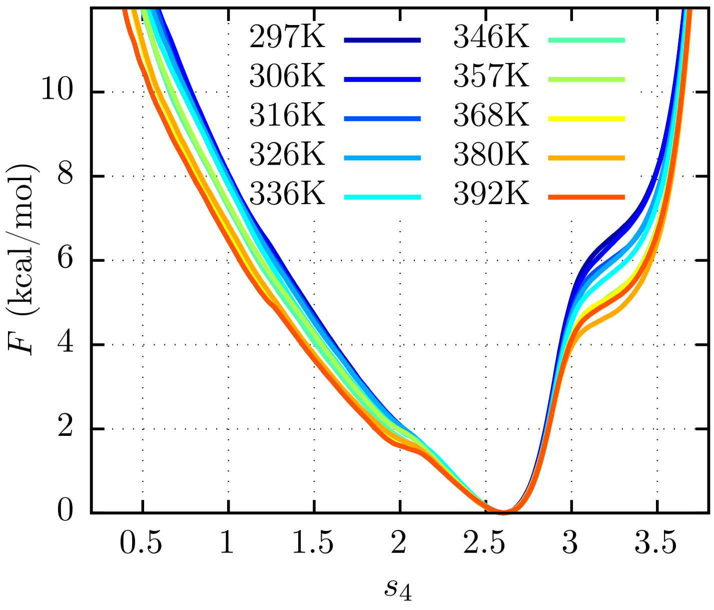

Figure S9: Average FEL obtained at different temperatures for the MoPrP.

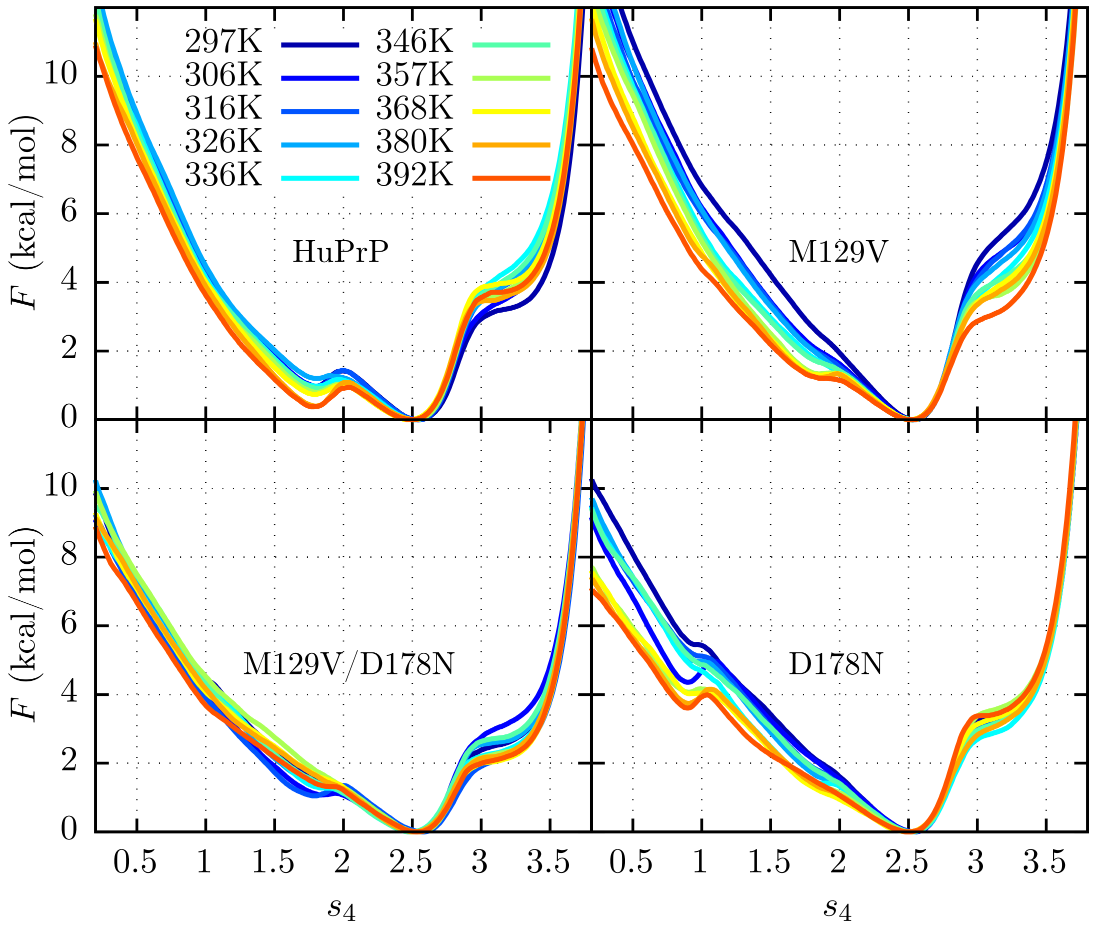

Figure S10: Average FEL obtained at different temperatures for the two 129-polymorph of HuPrP and its mutant D178N.

7 H-bond network at $s_4 \approx 1.8$

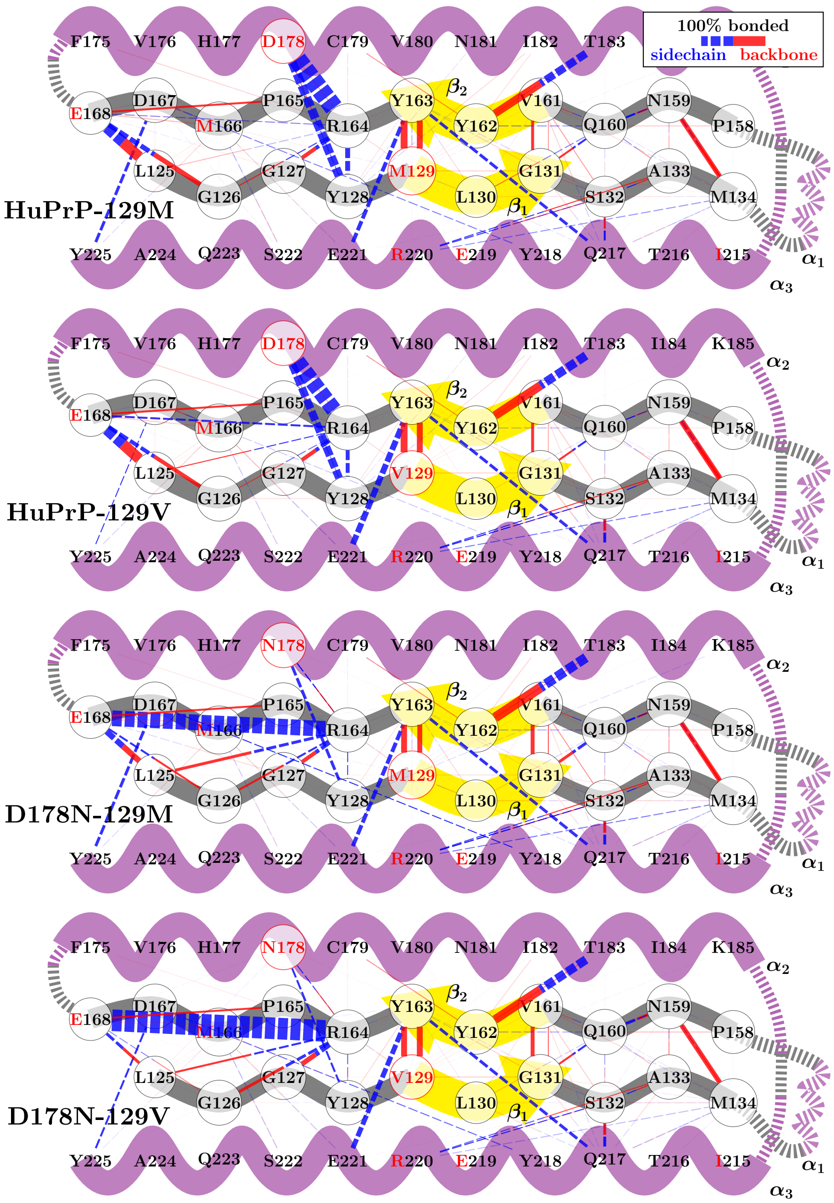

Figure S11: Schematic of the H-bondnetwork near the β-sheet structure of WT HuPrP and the D178N mutant. Bond thicknesses indicate relative frequencies with which bond lengths below 1.8 Å are observed at 309K.
